# Sex-dimorphic neuroestradiol regulation of ventromedial hypothalamic nucleus glucoregulatory transmitter and glycogen metabolism enzyme protein expression in the rat

**DOI:** 10.1186/s12868-020-00598-w

**Published:** 2020-11-25

**Authors:** Md. Main Uddin, Mostafa M. H. Ibrahim, Karen P. Briski

**Affiliations:** Willis-Knighton Endowed Professor of Pharmacy and Director, School of Basic Pharmaceutical and Toxicological Sciences, College of Pharmacy, University of Louisiana Monroe, 356 Bienville Building, 1800 Bienville Drive, Monroe, LA 71201 USA

**Keywords:** Letrozole, Aromatase, Insulin-induced hypoglycemia, Ventromedial hypothalamic nucleus, Glutamate decarboxylase_65/67_, Neuronal nitric oxide synthase

## Abstract

**Background:**

Ventromedial hypothalamic nucleus (VMN) gluco-regulatory transmission is subject to sex-specific control by estradiol. The VMN is characterized by high levels of aromatase expression.

**Methods:**

The aromatase inhibitor letrozole (LZ) was used with high-resolution microdissection/Western blot techniques to address the hypothesis that neuroestradiol exerts sex-dimorphic control of VMN neuronal nitric oxide synthase (nNOS) and glutamate decarboxylase_65/67_ (GAD) protein expression. Glycogen metabolism impacts VMN nNOS and GAD profiles; here, LZ treatment effects on VMN glycogen synthase (GS) and phosphorylase brain- (GPbb; glucoprivic-sensitive) and muscle (GPmm; norepinephrine-sensitive) variant proteins were examined.

**Results:**

VMN aromatase protein content was similar between sexes. Intracerebroventricular LZ infusion of testes-intact male and ovariectomized, estradiol-replaced female rats blocked insulin-induced hypoglycemic (IIH) up-regulation of this profile. LZ exerted sex-contingent effects on basal VMN nNOS and GAD expression, but blocked IIH-induced NO stimulation and GAD suppression in each sex. Sex-contingent LZ effects on basal and hypoglycemic patterns of GPbb and GPmm expression occurred at distinctive levels of the VMN. LZ correspondingly down- or up-regulated baseline pyruvate recycling pathway marker protein expression in males (glutaminase) and females (malic enzyme-1), and altered INS effects on those proteins.

**Conclusions:**

Results infer that neuroestradiol is required in each sex for optimal VMN metabolic transmitter signaling of hypoglycemic energy deficiency. Sex differences in VMN GP variant protein levels and sensitivity to aromatase may correlate with sex-dimorphic glycogen mobilization during this metabolic stress. Neuroestradiol may also exert sex-specific effects on glucogenic amino acid energy yield by actions on distinctive enzyme targets in each sex.

## Background

Estradiol controls diverse brain functions, including reproductive physiology and behavior, metabolic homeostasis, learning, and memory, through integrated activation of classical nuclear [estrogen receptor-alpha (ERα); estrogen receptor-beta (ERβ)] and membrane estrogen receptor [G-protein-coupled ER (GPER1/GPR30)] signaling [15, 29]. Central ERs are stimulated by estradiol of systemic and local origin. Neuroestradiol is generated in the brain by aromatase enzyme-catalyzed conversion from testosterone. Aromatase protein expression varies within the brain, with highest tissue content observed in distinct structures that include the medial preoptic area, bed nucleus of the *stria terminalis*, ventromedial hypothalamic nucleus (VMN), and medial amygdala [22, 27, 31]. Aromatase is implicated in male socio-sexual behavior [33] and female sexual behavior and reproductive neuroendocrine function [12, 19, 20]. There is credible support for the premise that acute regulatory effects of estradiol on behavior may reflect, in part, rapid aromatase-mediated adjustments in local brain tissue neuroestradiol concentrations that trigger non-genomic ER actions [3].

Research involving rat experimental models has revealed the VMN to be a critical component of the brain glucoregulatory network where nutrient, endocrine, and neurochemical cues are processed to shape glucose counter-regulation. VMN nitrergic and γ-aminobutyric acid (GABA)-ergic neurons express the ultra-sensitive energy sensor 5′-AMP-activated protein kinase (AMPK) [7, 17], and operate within local VMN circuitries to respectively stimulate or inhibit counter-regulatory outflow. Recent studies show that ERα and ERβ exert sex-contingent control of VMN expression of neuronal nitric oxide synthase (nNOS) and glutamate decarboxylase_65/67_ (GAD), marker proteins for nitric oxide and GABA transmission, during insulin-induced hypoglycemia [23]. The role of aromatase in glucostatic or glucoprivic patterns of VMN metabolic transmitter signaling is not known for either sex. Here, techniques for intracerebroventricular (*icv*) infusion of the aromatase inhibitor letrozole (LZ) were used in conjunction with high-resolution microdissection/Western blot techniques in a rat whole-animal model to address the hypothesis that neuroestradiol exerts sex-dimorphic control of VMN nNOS and/or GAD protein expression during eu- and/or hypoglycemia.

The complex carbohydrate glycogen is stored in astrocytes as a vital brain metabolic fuel reserve [32]. Glucose taken up from the circulation into these glia is either assembled into glycogen or converted to the oxidizable metabolic fuel l-lactate for trafficking to neurons [21]. Induction of lactoprivation within the mediobasal hypothalamus elevates counter-regulatory hormone secretion [6] by mechanisms involving diminished GABA signaling [11]. Glycogen metabolism is governed by opposing glycogen synthase (GS) and glycogen phosphorylase (GP) enzyme actions that catalyze glycogen synthesis or breakdown, respectively. Brain glycogenolysis, which is mediated by glycogen phosphorylase (GP) enzyme activity, increases under circumstances of insufficient energy supply versus demand, e.g. seizure, sleep deprivation, and hypoglycemia [9, 14] to liberate glucosyl units for conversion to l-lactate [5, 32]. Multiple GP isoforms are expressed in brain, including muscle- (GPmm) and brain- (GPbb) types, which differ with respect to cell-type localization and regulation by phosphorylation and AMP [25]. GPmm and -bb are both expressed in astrocytes, whereas GPbb occurs exclusively in neurons. Phosphorylation elicits complete versus partial activation of GPmm or GPbb, whereas GPbb exhibits greater affinity for and sensitivity to AMP activation relative to GPmm, and requires AMP binding for optimal enzyme function and Km. Recent studies show that inhibition of total VMN GP activity up-regulates VMN nNOS expression in each sex ([1]; Alshamrani and Briski, personal communication), and suppresses glutamate decarboxylase_65/67_ (GAD) profiles in females [Alshamrani and Briski, personal communication], inferring that reduced astrocyte glycogen mass or turnover may signal energy shortage to VMN gluco-regulatory neurons. This research investigated whether LZ pretreatment alters eu- or hypoglycemic patterns of VMN GS, glucoprivic-sensitive GP-brain type (GPbb), and norepinephrine (NE)-sensitive GP-muscle type (GPmm) protein expression in a sex-specific manner.

There is emerging evidence that brain neurons may adapt to hypoglycemia by utilizing non-glucose – derived energy fuels. Glutamine (Gln) and glutamate (Glu) are potential cerebral energy substrates during glucoprivation as brain levels of these amino acids decline during hypoglycemia [4]. Glutaminolysis is an energy-yielding pathway that utilizes glutamine metabolism, via conversion of Gln to Glu, within the pyruvate recycling pathway [10] to support tricarboxylic acid (TCA) cycle function. Complete glutamine oxidation in the TCA cycle involves entrance and exit of glutamate in the form of alpha-ketoglutarate and malate, respectively, followed by processing of pyruvate to acetyl-CoA. Current studies examined the premise that neuroestradiol may regulate expression of the rate-limiting pyruvate recycling pathway enzymes glutaminase (GLT) and NADP-dependent malic enzyme-1 (ME-1) in the male and/or female rat VMN during glucostasis or glucoprivation.

## Materials and methods

### Animals

Adult male (M; average age: 3 months ± 6 days; body weight: 394 ± 32 g) and female (F; average age: 3.5 months ± 7 days; body weight: 265 ± 31 g) Sprague–Dawley rats (Envigo, Houston, TX) were housed in groups of 2–3 in shoe-box cages containing Aspen Sani chip bedding (Envigo), according to sex. Animals were maintained under a 14 h light/10 h dark cycle (lights on at 05.00 h), allowed ad libitum access to standard laboratory chow (prod. no. Harlan Teklad LM-485; Harlan Industries, Madison, WI) and tap water, and acclimated to daily handling before experimentation. All surgical and experimental protocols were conducted in accordance with the National Institutes of Health Guide for the Care and Use of Laboratory Animals, 8th Edition, under approval by the ULM Institutional Animal Care and Use Committee (protocol no. 19AUG-KPB-01)

### Experimental design

Animals of each sex (male: M; female: F) were randomly assigned to one of four treatment groups (Table 1). All surgeries were performed in a dedicated surgical suite in the College of Pharmacy Vivarium, adjacent to housing quarters. Animal health and welfare status was checked daily by investigators and Vivarium management over the duration of the experiment. On day 1, female rats were bilaterally ovariectomized (OVX) under ketamine/xylazine anesthesia (0.1 mL/100 g bw; 90 mg ketamine: 10 mg xylazine/mL; Henry Schein Inc., Melville, NY) and implanted with a subcutaneous (*sc*) silastic capsule (i.d. 0.062 inch, o.d. 0.125 inch; 10 mm/100 g bw) containing 30 μg 17β estradiol-3-benzoate/mL safflower oil. This steroid replacement regimen yields approximate plasma estradiol concentrations of 22 pg/mL [8], which replicate circulating hormone levels measured in ovary-intact, four-day cycling female rats at metestrus. On day 5, male and female animals were implanted with a 28-gauge stainless-steel cannula (Alzet Brain Infusion Kit 1, DURECT™ Corporation, Cupertino, CA) aimed at the left lateral ventricle (LV) [coordinates: 0.0 mm posterior to *bregma*; 1.5 mm lateral to *bregma*; 3.5 mm ventral to brain surface], connected to a primed Model 1007D Alzet osmotic minipump (0.5 μL/h) filled with vehicle [V; 30% artificial cerebrospinal fluid (aCSF)/70% dimethyl sulfoxide (DMSO); groups V/V-M; V/INS-M; V/V-F/V/INS-F; n = 6 per group] or Lz (1.67 μg/μL [26]; prod. no. L0248; Tokyo Chemical Industries, Tokyo, Japan; groups Lz/V-M; Lz/INS-M; Lz/V-F; Lz/INS-F; n = 6 group). G*Power statistical a priori power analysis predicted power = 0.80 at this sample size. After surgery, animals were injected *sc* with ketoprofen (1 mg/kg bw) and intramuscularly with enrofloxacin (10 mg/0.1 mL), then transferred to individual cages. At 10 min intervals between 9.00 and 9.20 h on day 11, rats were selected by random order from a single treatment group for *sc* injection of sterile insulin diluent (V; Eli Lilly & Co., Indianapolis, IN) or neutral protamine Hagedorn insulin (INS, 10.0 U/kg bw; Henry Schein), in the absence of anesthesia. To maintain uniform timing between *sc* injection and sacrifice (+1 h) throughout the experiment, animals were anesthetized with isoflurane at 10.00, 10.10, or 10.20 h for blood collection by cardiac puncture, then immediately sacrificed by focused microwave fixation (1.45 s; In Vivo Microwave Fixation System, 4.5 kW; Stoelting Co., Wood Dale, IL). All subjects were euthanized; none were released. Focused microwave fixation is optimal for preservation of brain glycogen as instantaneous heat induction halts metabolizing enzyme activity. No animals were excluded from the study due to health complications. Brains were snap-frozen in a liquid nitrogen-cooled isopentane for storage at − 80 °C. Plasma was stored at − 20 °C.

**Table 1:** Experimental design

	*Icv*^a^ Pretreatment	Subcutaneous injection	Group identifier	
	Day 5-Day	Day 11		
Treatment groups				
Male	V^b^	V^c^	V/V-M	n = 6
	V	INS^d^	V/INS-M	n = 6
	Lz^e^	V	Lz/V-M	n = 6
	Lz	INS	Lz/INS-M	n = 6
Female	V	V	V/V-F	n = 6
	V	INS	V/INS-F	n = 6
	Lz	V	Lz/V-F	n = 6
	Lz	INS	Lz/INS-F	n = 6

^a^ Left lateral ventricle

^b^ Artificial cerebrospinal fluid (30%)/dimethyl sulfoxide (70%)

^c^ Sterile diluent; 100 μL/100 g bw

^d^ 10.0 U neutral protamine Hagedorn insulin/kg bw

^e^ 1.67 μg/μL dosage, 0.5 μL/h infusion rate, 7 day duration of pretreatment

### VMN tissue micropunch dissection and western blot analysis

Each brain was cut into consecutive 100 μm-thick frozen sections through the VMN between − 2.00 and − 3.3 mm posterior to *bregma*. Bilateral micropunches of VMN tissue taken from sections cut at rostral (− 2.0 to − 2.3 mm), middle (− 2.5 to − 2.8), and caudal (− 3.0 to − 3.3 mm) levels of the VMN were pooled according to region in lysis buffer [16] for Western blot analysis. Within each treatment group, heat-denatured tissue aliquots from each subject were combined within rostral, middle, and caudal VMN regions to create sextuplicate sample pools for each protein of interest. Tissue sample pools were separated in Bio-Rad TGX 10% stain-free gels (Bio-Rad, Hercules, CA); after electrophoresis, gels were UV light-activated (1 min) in a Bio-Rad ChemiDoc TM Touch Imaging System [16] and proteins were transblotted to 0.45-μm PVDF-Plus membranes (prod. no. 121,639; Data Support Co., Panorama City, CA). Membranes were blocked with Tris-buffer saline containing 0.1% Tween-20 and 2.0% bovine serum albumin prior to incubation between 36 and 42 h (4 °C) with primary polyclonal antisera raised in rabbit against GS (1:2000; prod. no. 3893S; Cell Signaling Technology, Danvers, MA), GPbb (1:,2000; prod. no. NBP1-32799; Novus Biologicals, Littleton, CO), GPmm (1:2000; prod. No. NBP2-16689; Novus Biol.), GAD (1:10,000; prod. no. ABN904; Millipore Sigma, Burlington, MA), nNOS (1:1500; prod. no. NBP1-39681; Novus Biol.), glutaminase (GLT; 1:1500; prod. no. NBP1-89766; Novus Biol.), malic enzyme 1 (ME1; 1:2000; prod. no. NBP1-32398; Novus Biol.) or aromatase (1:2000, prod. no. NB100-1596, Novus Biol.) (Table 2). Membranes were next incubated with a goat anti-rabbit secondary antiserum (1:5000; prod. no. NEF812001EA; PerkinElmer, Waltham, MA), then SuperSignal West Femto maximum sensitivity chemiluminescence substrate (prod. no. 34096; ThermoFisherScientific, Waltham, MA). Automated membrane buffer washes and blocking and antibody incubations were performed in a Freedom Rocker™ Blotbot. Protein band optical density (O.D.) measures were normalized to total in-lane protein using Image Lab™ 6.0.0 software (Bio-Rad). Precision plus protein molecular weight dual color standards (prod. no. 161-0374, Bio-Rad) were included in each Western blot analysis.

**Table 2:** Description of primary antisera used

Antibody	Manufacturer	Product number	RRID	Application/Conc.	Antigen sequences	References	PMID
Aromatase	Novus Biologicals LLC, Centennial CO, US	NB100-1596	AB_10000919	WB; 1:2000 (0.5 μL/mL)	Human Aromatase protein (between residues 400-502). [UniProt# P11511]	Giles et al., Breast Cancer Res. 2018; 20(1): 50	29898754
Neuronal Nitric oxide synthase (nNOS)	Novus Biologicals	NBP1-39681	AB_2282822	WB; 1:1500 (0.75 μL/mL)	Human nNOS amino acids 1422-1433 (ESKKDTDEVFSS)	Mahmood et al., Mol. Cell. Neurosci. 2019; 95: 51-58	30660767
Glutamate Decarboxylase 65/67 (GAD)	Millipore Sigma, Burlington, MA, US	ABN904	Not available	WB; 1:10000 (0.1 μL/mL)	KLH-conjugated linear peptide corresponding to 14 amino acids from the C-terminal region of human glutamate decarboxylase 2 (GAD2).	Ibrahim, et al., Brain Res. 2019; 1711: 48-57	30629946
Glycogen Phosphorylase-Brain Type (GPbb)	Novus Biologicals	NBP1-32799	AB_2253353	WB; 1:2000 (0.5 μL/mL)	Recombinant protein encompassing a sequence within the C-terminus region of human GPBB. The exact sequence is proprietary	Ali MH et al.; Neuroscience 2019; 409: 253-260	30954669
Glycogen Phosphorylase-Muscle Type (GPmm)	Novus Biologicals	NBP2-16689	Not available	WB; 1:2000 (0.5 μL/mL)	Recombinant protein encompassing a sequence within the center region of human PYGM. The exact sequence is proprietary	Ali MH et al.; Neuroscience 2019; 409: 253-260	30954669
Glycogen Synthase (GS)	Cell Signaling Technology LLC, Danvers CT, US	3893S	AB_2279563	WB; 1:2000 (0.5 μL/mL)	Antibody detects total muscle glycogen synthase protein	Ibrahim et al., Brain Res. 2019; 1711: 48-57	30629946
Glutaminase (GLT)	Novus Biologicals	NBP1-89766	AB_11013964	WB; 1:1500 (0.75 μL/mL	DRWNNTPMDEALHFGHHDVFKILQEYQVQYTPQGDSDNGKENQTVHKNLDGL	Uddin et al., Brain Res. 2019; 1720: 146311	31265816
Malic Enzyme 1 (ME1)	Novus Biologicals	NBP1-32398	AB_2143825	WB; 1:2000 (0.5 μL/mL)	Recombinant protein encompassing a sequence within the center region of human ME1. The exact sequence is proprietary	Uddin et al., Brain Res. 2019; 1720: 146311	31265816

### Plasma glucose analysis

Circulating glucose levels was measured with an ACCU-CHECK Aviva Plus glucometer (Roche Diagnostics USA, Indianapolis, IN), as described [18].

### Statistical analyses

Mean normalized tissue Western blot protein O.D. and plasma glucose measures were evaluated using three-way analysis of variance and Student–Newman–Keuls post hoc test. Differences of p < 0.05 were considered significant. In each figure, statistical differences between specific pairs of treatment groups are denoted with the following symbols: *p < 0.05; **p < 0.01; ***p < 0.001; ****p < 0.0001.

## Results

**Fig. 1:**
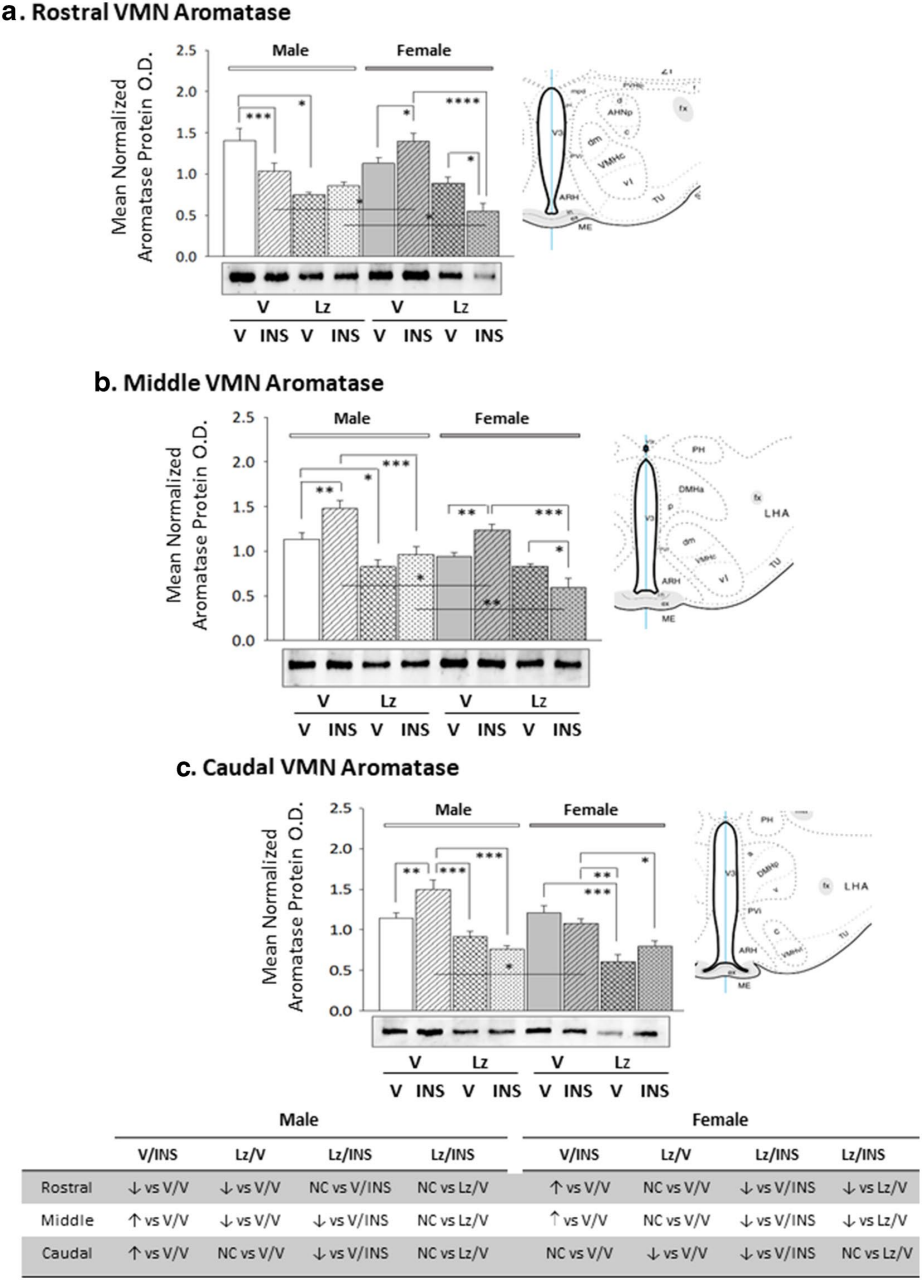
Effects of lateral ventricular (LV) infusion of the aromatase enzyme inhibitor letrozole (Lz) on basal and hypoglycemic patterns of ventromedial hypothalamic nucleus (VMN) aromatase protein expression in male versus female rats. VMN tissue was micropunch-dissected over predetermined rostro-caudal levels from adult testes-intact male and estradiol-implanted, ovariectomized female rats pretreated by LV Lz infusion before subcutaneous (sc) insulin (INS) or vehicle (V) injection. Data depict mean normalized rostral (**a**; F_(7,40)_ = 11.73; p < 0.0001), middle (**b**; F_(7,40)_ = 14.14; p < 0.0001), and caudal (**c**; F_(7,40)_ = 13.50; p < 0.0001) VMN aromatase protein optical density (O.D.) measures ± S.E.M. for groups of male (M; *left*-*hand* side; solid or patterned white bars) and female (F; right-hand side; solid or patterned gray bars) rats treated as follows: V infusion/V injection (V/V-M, solid white bars, n = 6; V/V-F, solid gray bars; n = 6), V infusion/INS injection (V/INS-M; diagonal-striped white bars; n = 6; V/INS-F; diagonal-striped gray bars; n = 6), Lz infusion/V injection (Lz/V-M; cross-hatched white bars; n = 6; Lz/V-F; cross-hatched gray bars, n = 6), Lz infusion//INS injection (Lz/INS-M; stippled white bars; n = 6; Lz/INS-F; stippled gray bars; n = 6). *p < 0.05; **p < 0.01; ***p < 0.001; ****p < 0.0001. The table presented at bottom summarizes differences (↑, increase; ↓, decrease) or no change (NC) in rostral, middle, and caudal VMN aromatase protein expression between treatment groups in each sex

Figure 1 illustrates effects of intra-LV infusion of the aromatase enzyme inhibitor letrozole (Lz) to adult male and female rats on basal and insulin-induced hypoglycemic (IIH) patterns of VMN aromatase protein expression. Data reveal that in each sex, Lz caused VMN region-specific adjustments in aromatase expression as Lz suppressed rostral (Panel A) and middle (Panel B) VMN protein content in males [Lz/V-M versus V/V-M], but decreased aromatase levels in the female caudal VMN [Lz/V-F versus V/V-F] (Panel C). Insulin dosing of male rats elicited an Lz-preventable [Lz/INS-M versus V/INS-M] decline (rostral VMN) or intensification (middle and caudal VMN) of aromatase expression [V/INS-M versus V/V-M], according to VMN rostro-caudal level. On the other hand, female animals injected with insulin exhibited augmented rostral and middle, but not caudal VMN aromatase protein levels [V/INS-F versus V/V-F]; hypoglycemic up-regulation of this profile was averted at each VMN level by Lz [Lz/INS-F versus V/INS-F]. As shown in Table 3, plasma glucose levels were significantly decreased in either sex in response to INS injection; this response was not impaired by Lz. Full-length blots of aromatase and other target proteins are presented as Additional file 1.

**Table 3:** Effects of letrozole (Lz) pretreatment on plasma glucose levels in vehicle (V)- or insulin (INS)-injected male and female rats

Sex	Treatment groups^e^			
	V/V^a^	V/INS^b^	Lz/V^c^	Lz/INS^d^
Male	174.5 ± 11.1	75.3 ± 4.1*	182.3 ± 8.9	72.8 ± 2.3*
Female	169.7 ± 5.9	60.3 ± 3.8*	148.2 ± 7.1***	79.5 ± 4.5**

^a^ Intracerebroventricular (*icv*) V infusion days 5–11; subcutaneous (*sc*) V injection day 11

^b^ *icv* V infusion days 5–11; *sc* neutral protamine Hagedorn insulin (INS; 10 U/kg bw) day 11

^c^ *icv* letrozole (Lz; 1.67 µg/µL dosage, 0.5 µL/h infusion rate) infusion days 5–11; *sc* V injection day 11

^d^ *icv* Lz infusion days 5–11; *sc* INS injection day 11

^e^ F_(7/40)_ = 68.61; *p *< 0.0001

* p < 0.0001 versus V-injected controls

** p < 0.005 versus V-injected controls

*** p < 0.001 Lz/V-M versus Lz/V-F

**Fig. 2:**
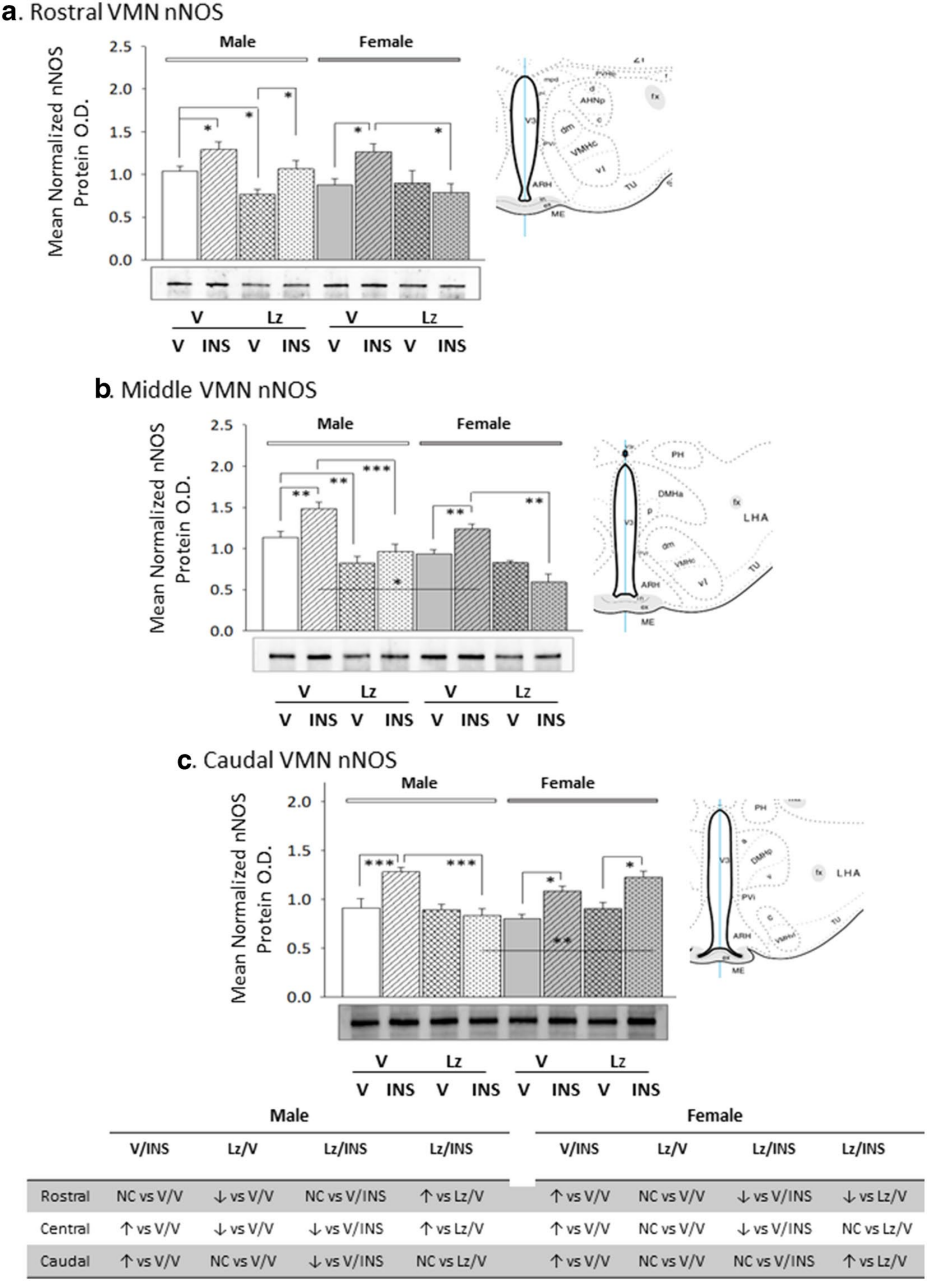
Expression profiles of the nitrergic signal marker protein neuronal nitric oxide synthase (nNOS) in male versus female rat VMN during eu- and hypoglycemia; impact of intracerebroventricular (*icv*) Lz infusion. Mean normalized rostral (**a**; F_(7,40)_ = 4.64; p = 0.001), middle (**b**; F_(7,40)_ = 7.86; p = 0.001), and caudal (**c**; F_(7,40)_ = 10.04; p < 0.0001) VMN nNOS protein O.D. values ± S.E.M. are presented for V/V, V/INS, Lz/V, and Lz/INS groups of male (M; *left*-*hand* side) and female (F; *right*-*hand* side) rats; n = 6 animals per group. *p < 0.05; **p < 0.01; ***p < 0.001; ****p < 0.0001. Results are summarized in the table at bottom; ↑ and ↓ denote a relative increase or decrease, respectively, between treatment groups; no change (NC) between groups is also indicated

**Fig. 3:**
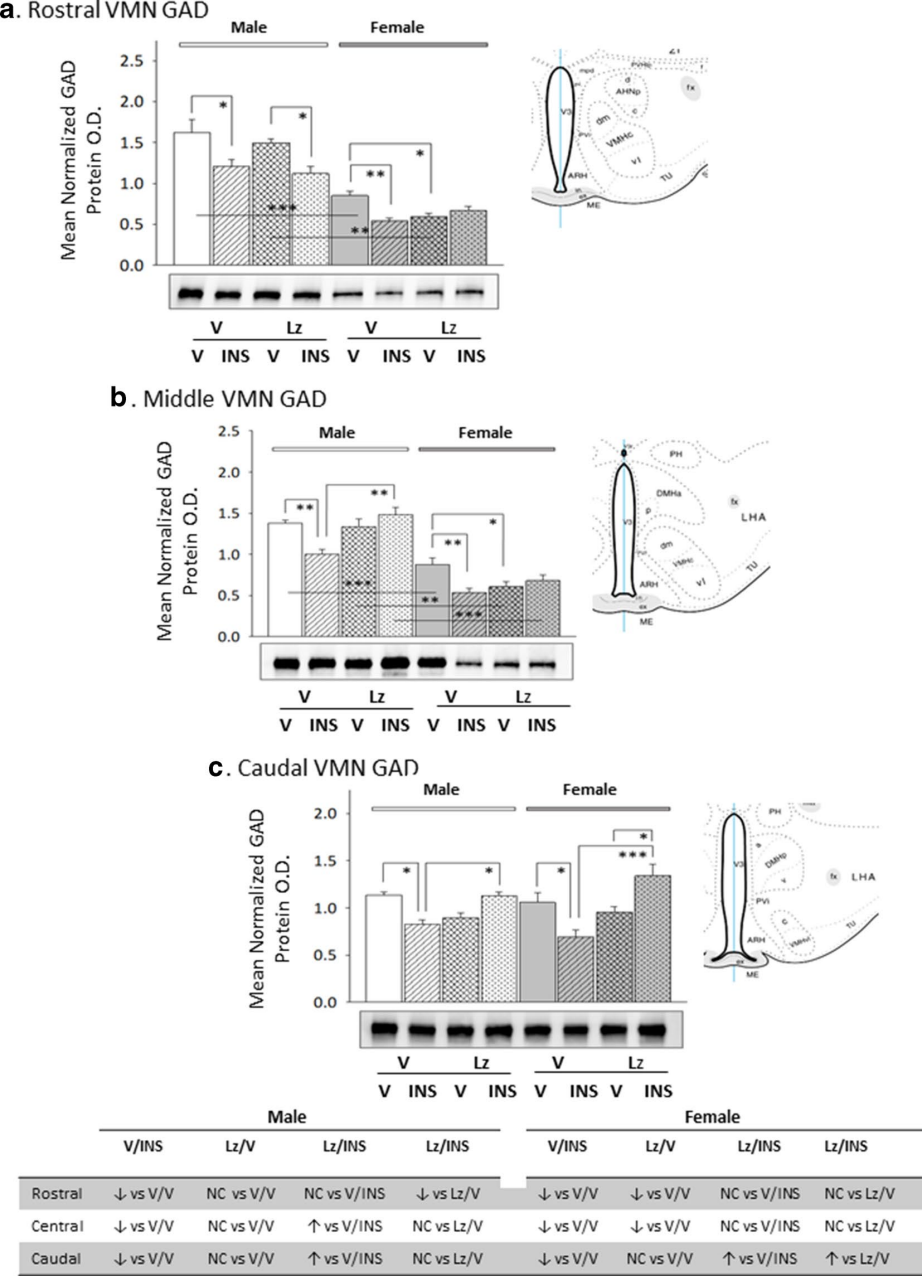
Basal and hypoglycemic patterns of VMN gluco-inhibitory γ-aminobutyric acid (GABA) marker protein expression in *icv* Lz-pretreated male versus female rats. The GABA transmitter marker protein glutamate decarboxylase_65/67_ (GAD) was measured in the rostral (**a**; F_(7,40)_ = 27.15; p < 0.0001), middle (**b**; F_(7,40)_ = 28.85; p < 0.0001), and caudal (**c**; F_(7,40)_ = 8.07; p < 0.0001) VMN of V/V, V/INS, Lz/V, and Lz/INS groups of male (M; left-hand side) and female (F; right-hand side) rats. *p < 0.05; **p < 0.01; ***p < 0.001; ****p < 0.0001. For each VMN segment, data depict mean normalized GAD O.D. values ± S.E.M. Results are summarized in the table at bottom; ↑ and ↓ denote a relative increase or decrease, respectively, between treatment groups; no change (NC) between groups is also indicated

Data in Fig. 2 depict *icv* Lz regulation of VMN nNOS protein expression in eu- versus hypoglycemic male and female rats. Outcomes reveal sex-dimorphic Lz effects on baseline nNOS as Lz diminished male rostral and middle VMN nNOS content, but did not alter this protein profile at any level of the female VMN. In both sexes, insulin therapy elevated nNOS protein levels in each of the three VMN region examined. Deterrence of insulin stimulation of VMN nNOS by Lz was region-specific, as efficacy of this pretreatment was observed in the middle and caudal VMN in males or in the rostral and middle VMN in the female. Figure 3 depicts patterns of VMN GAD protein expression in Lz-pretreated male and female rats under eu- or hypoglycemic conditions. Rostral and middle VMN GAD content was higher in euglycemic males versus females [V/V-M versus V/V-F]. Data show that Lz suppressed GAD profiles in the female VMN, namely at rostral and middle levels, but did not alter male VMN GAD content. In each sex, insulin administration caused a decline in GAD content in each VMN region investigated. Lz pretreatment abolished this inhibitory response to insulin in the male middle and caudal VMN, but effectively prevented adjustments in GAD expression at each level of the female VMN.

**Fig. 4:**
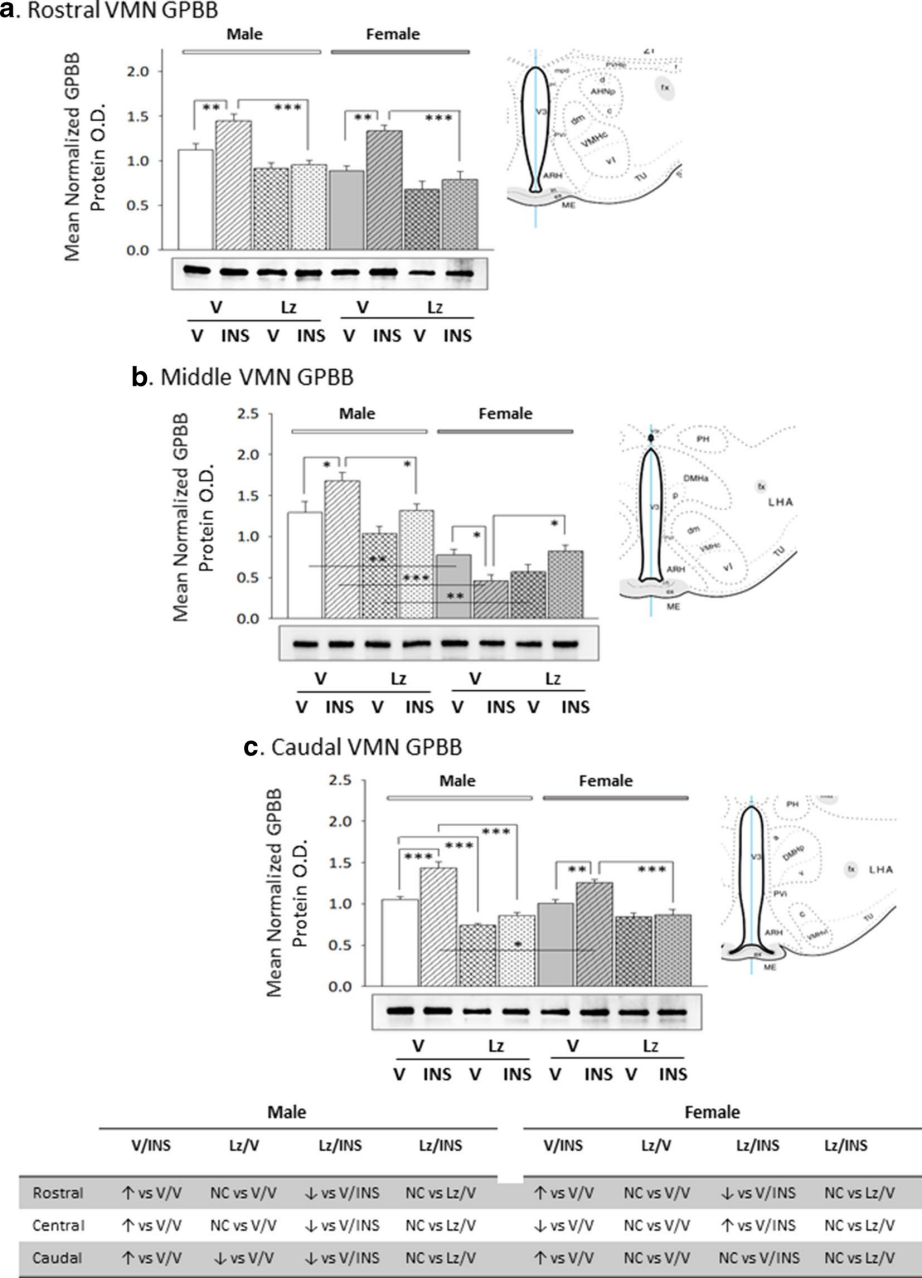
Expression profiles of glycogen synthase (GS) in male versus female rat VMN; impact of *icv* Lz infusion. Mean normalized rostral (**a**; F_(7,40)_ = 4.93; p = 0.001), middle (**b**; F_(7,40)_ = 6.60; p = 0.001), and caudal (**c**; F_(7,40)_ = 5.51; p < 0.0001) VMN GS protein O.D. values ± S.E.M. are presented for V/V, V/INS, Lz/V, and Lz/INS groups of male (M; left-hand side) and female (F; right-hand side) rats; n = 6 animals per group. *p < 0.05; **p < 0.01; ***p < 0.001; ****p < 0.0001. Results are summarized in the table *at bottom*; ↑ and ↓ denote a relative increase or decrease, respectively, between treatment groups; no change (NC) between groups is also indicated

**Fig. 5:**
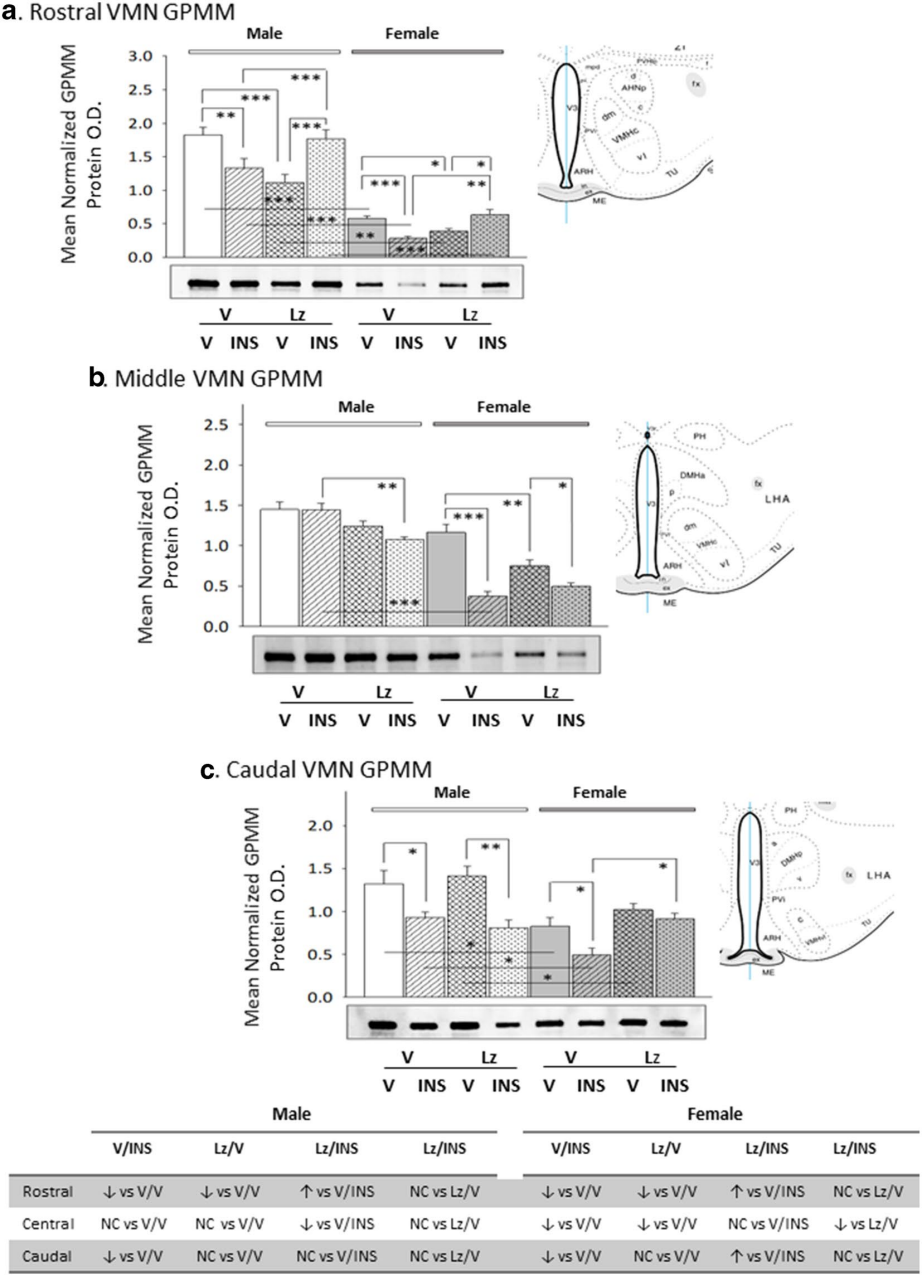
Region-based patterns of glycogen phosphorylase-muscle type (GPmm) Protein expression in *icv* Lz-pretreated male and female rats. GPmm protein was measured by Western blot in the rostral (**a**; F_(7,40)_ = 35.87; p < 0.0001), middle (**b**; F_(7,40)_ = 33.89; p < 0.0001), and caudal (**c**; F_(7,40)_ = 9.43; p < 0.0001) VMN of V/V, V/INS, Lz/V, and Lz/INS groups of male (M; left-hand side) and female (F; right-hand side) rats. *p < 0.05; **p < 0.01; ***p < 0.001; ****p < 0.0001. For each VMN segment, data depict mean normalized GAD O.D. values ± S.E.M. Results are summarized in the table at bottom; ↑ and ↓ denote a relative increase or decrease, respectively, between treatment groups; no change (NC) between groups is also indicated

**Fig. 6:**
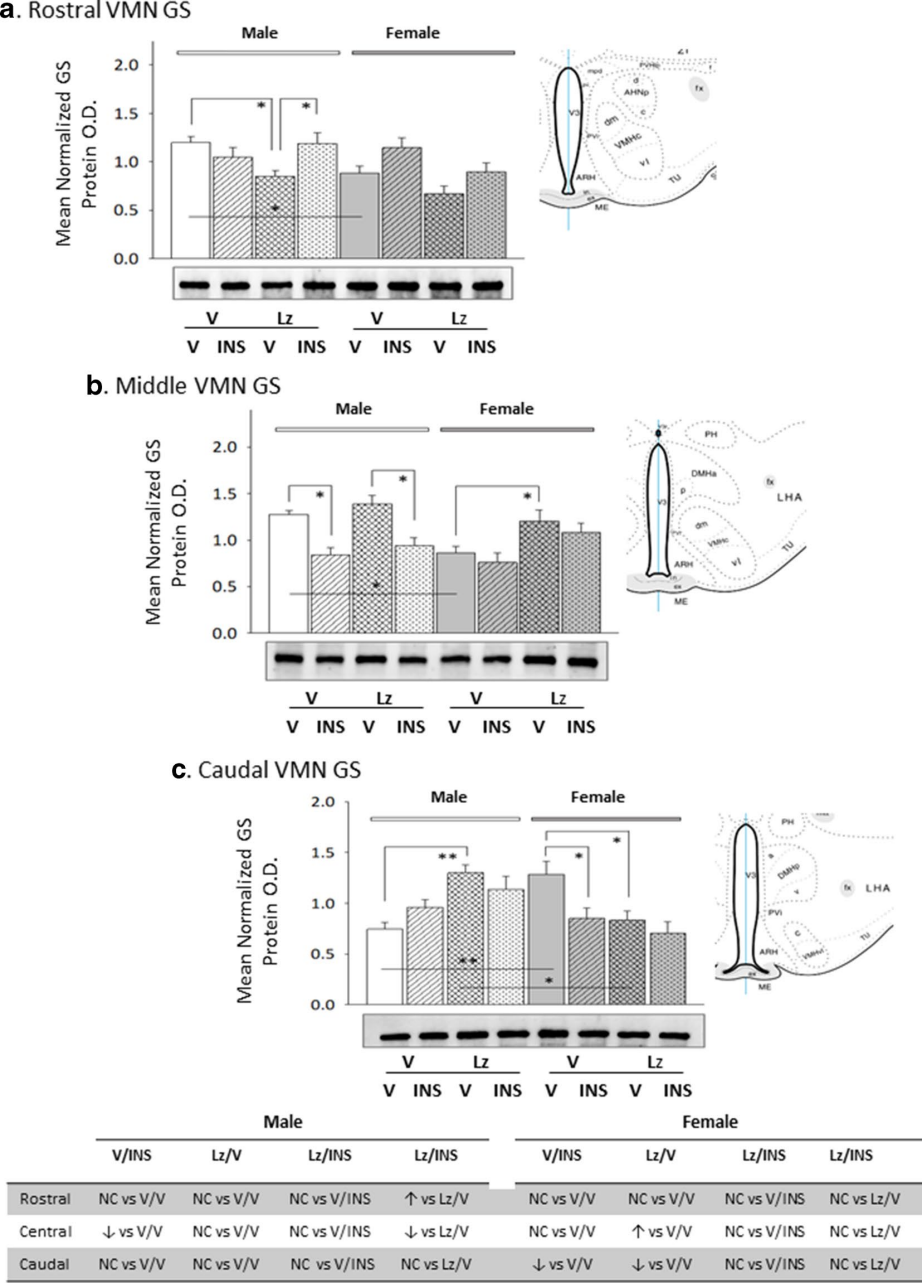
Basal and hypoglycemic expression profiles of glycogen phosphorylase-brain type (GPbb) in male versus female rat VMN; impact of *icv* Lz infusion. Mean normalized rostral (**a**; F_(7,40)_ = 14.20; p < 0.0001), middle (**b**; F_(7,40)_ = 20.97; p < 0.0001), and caudal (**c**; F_(7,40)_ = 25.06; p < 0.0001) VMN GPbb protein O.D. values ± S.E.M. are presented for V/V, V/INS, Lz/V, and Lz/INS groups of male (M; left-hand side) and female (F; right-hand side) rats; n = 6 animals per group. *p < 0.05; **p < 0.01; ***p < 0.001; ****p < 0.0001. Results are summarized in the table *at bottom*; ↑ and ↓ denote a relative increase or decrease, respectively, between treatment groups; no change (NC) between groups is also indicated

Figure 4 describes effects of *icv* Lz on basal and hypoglycemic patterns of VMN GS protein expression in male and female rats. Baseline VMN GS protein levels were greater in rostral and middle VMN region in males compared to females [V/V-M versus V/V-F], yet GS content was higher in the female versus male caudal VMN. Lz regulation of GS content was VMN region-specific adjustments in each sex, as this treatment diminished GS profiles in the rostral male VMN or caudal female VMN, but increased GS content at caudal or middle levels of the male or female VMN, respectively. Insulin suppressed GS protein expression in the middle region of the male VMN and in the caudal female VMN. Lz deterred hypoglycemic inhibition of VMN GS protein expression in female, but not male rats. Data presented in Fig. 5 show that baseline GPmm protein expression was relatively greater in the male rostral and caudal VMN compared to females. Outcomes disclose that Lz infusion diminished VMN GPmm profiles in the rostral male VMN and in the rostral and middle VMN in females. Insulin injection suppressed GPmm expression in the rostral and caudal VMN in males; this inhibitory response was averted by Lz in the rostral, but not caudal VMN. Hypoglycemia-associated suppression of GPmm protein was observed throughout the rostro-caudal extent of the female VMN; Lz abolished this decline in the rostral and caudal VMN, but not in the middle region. Figure 6 illustrates eu- versus hypoglycemic patterns of VMN GPbb protein expression in Lz-pretreated male and female rats. Baseline GPbb content was higher in the male middle VMN compared to females. Data indicate that Lz inhibited caudal VMN GPbb profiles in the males, but did not modify VMN GPbb content in female animals. Insulin injection increased GPbb expression throughout the male VMN; this stimulatory response was alleviated by Lz. Insulin-injected female rats exhibited amplified rostral and caudal VMN GPbb content, alongside a decline in this protein profile in the middle VMN.

**Fig. 7:**
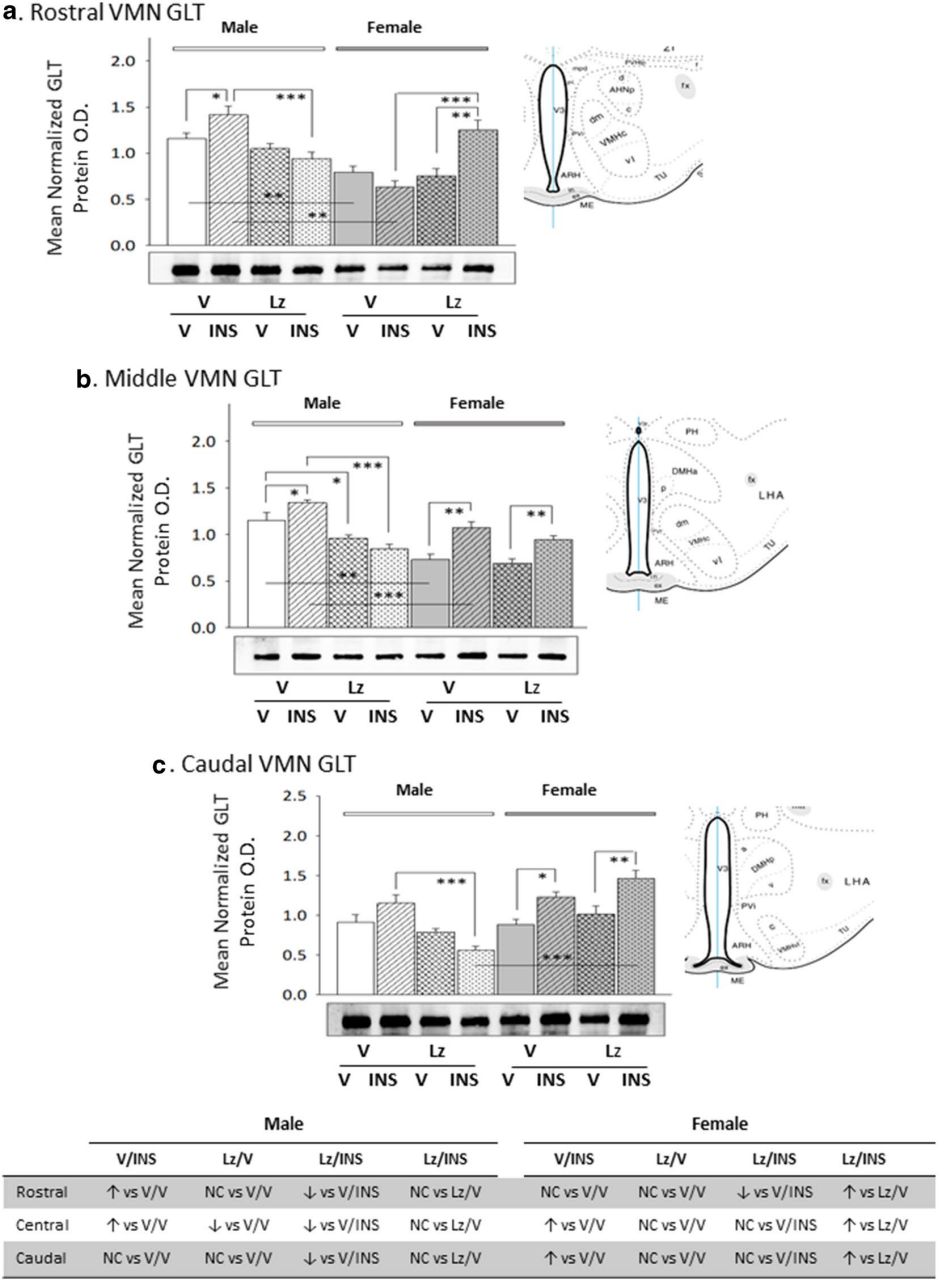
Region-based expression of the pyruvate recycling pathway marker protein glutaminase (GLT) the VMN of *icv* Lz-pretreated male and female rats. GLT protein was measured by Western blot in the rostral (**a**; F_(7,40)_ = 12.20; p < 0.0001), middle (**b**; F_(7,40)_ = 17.39; p < 0.0001), and caudal (**c**; F_(7,40)_ = 11.67; p < 0.0001) VMN of V/V, V/INS, Lz/V, and Lz/INS groups of male (M; *left*-*hand* side) and female (F; *right*-*hand* side) rats. *p < 0.05; **p < 0.01; ***p < 0.001; ****p < 0.0001. For each VMN segment, data depict mean normalized GAD O.D. values ± S.E.M. Results are summarized in the table *at bottom*; ↑ and ↓ denote a relative increase or decrease, respectively, between treatment groups; no change (NC) between groups is also indicated

**Fig. 8:**
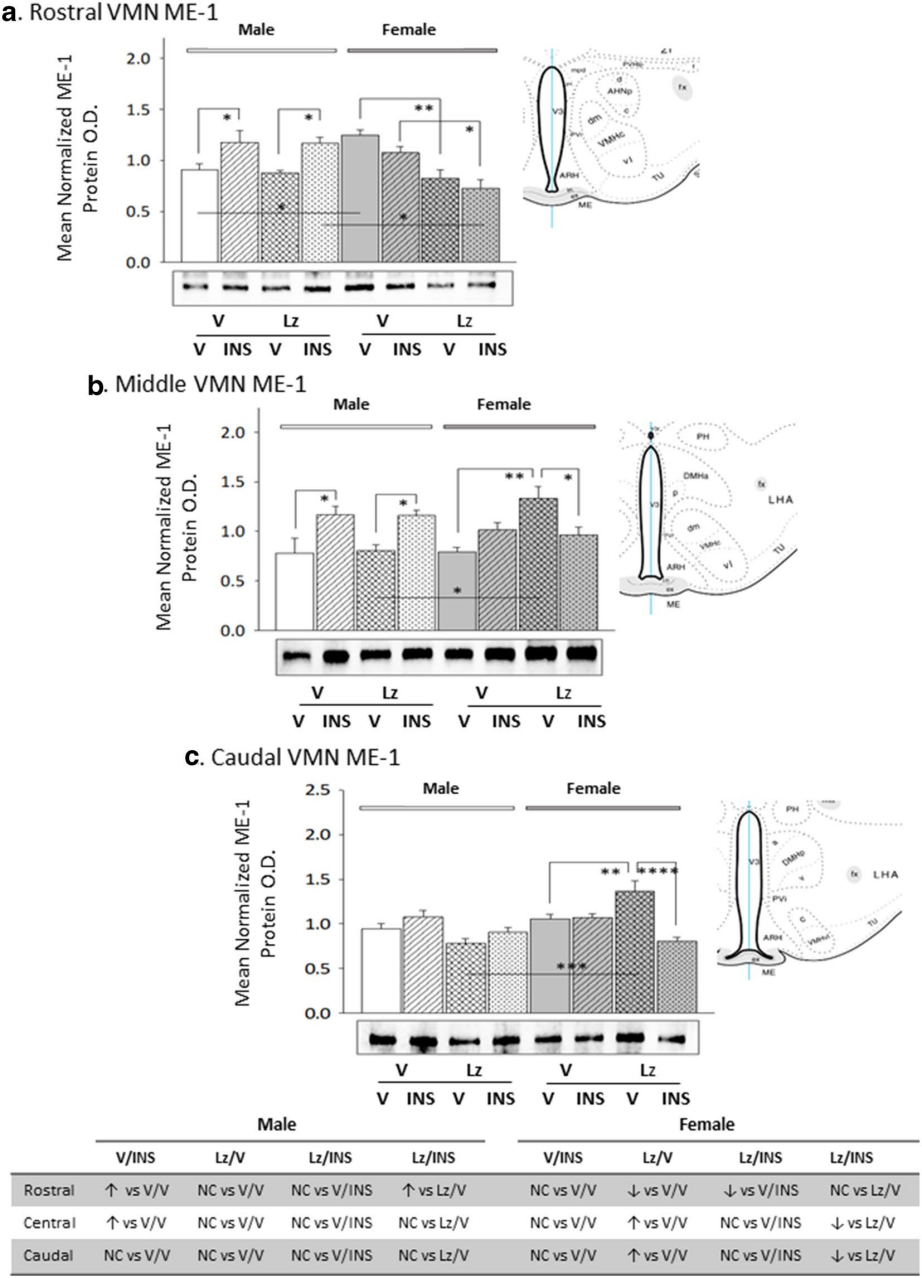
Expression profiles of pyruvate recycling pathway marker protein malic enzyme-1 (ME-1) protein in male versus female rat VMN; Impact of *icv* Lz infusion. Mean normalized rostral (**a**; F_(7,40)_ = 7.21; p < 0.0001), middle (**b**; F_(7,40)_ = 5.52; p < 0.0001), and caudal (**c**; F_(7,40)_ = 8.14; p < 0.0001) VMN GPbb protein O.D. values ± S.E.M. are presented for V/V, V/INS, Lz/V, and Lz/INS groups of male (M; left-hand side) and female (F; right-hand side) rats; n = 6 animals per group. *p < 0.05; **p < 0.01; ***p < 0.001; ****p < 0.0001. Results are summarized in the table *at bottom*; ↑ and ↓ denote a relative increase or decrease, respectively, between treatment groups; no change (NC) between groups is also indicated

Data presented in Fig. 7 depict Lz effects on VMN GLT protein expression in eu- versus hypoglycemic male and female rats. Baseline GLT content was higher in the male rostral and middle VMN compared to females. Lz suppressed baseline GLT profiles in the male VMN (middle region), but did not modify protein content in the female VMN. Insulin-injected male rats exhibited elevated GLT content in the rostral and middle VMN, whereas females showed GLT up-regulation in the middle and caudal VMN in response to that treatment. This stimulatory response was prevented by Lz in male, but not female rats. As shown in Fig. 8, basal ME-1 content was higher in the female rostral VMN compared to male rats. Lz did not affect baseline VMN ME-I protein expression in males, but either reduced (rostral VMN) or increased (middle and caudal VMN) this profile in females. Insulin treatment elevated ME-1 content of the male rostral and middle VMN; Lz did not prevent these stimulatory responses. Female rat VMN ME-1 profiles were refractory to insulin therapy.

## Discussion

The VMN is a prominent site of aromatase expression in the brain. Here, microdissected VMN tissue from male and female rats infused *icv* with the aromatase inhibitor LZ prior to *sc* vehicle or insulin injection was analyzed by Western blot to address the premise that forebrain-generated estradiol controls VMN glucoregulatory metabolic signaling, glycogen accumulation and mobilization, and utilization of alternative non-glucose energy fuels in a sex-specific manner. IIH elicited Lz-reversible, region-specific adjustments in VMN aromatase expression in each sex. LZ caused sex-dimorphic effects on VMN nNOS and GAD profiles in euglycemic animals, but prevented hypoglycemic effects on these protein profiles within distinct rostro-caudal VMN levels in each sex. Sex-contingent LZ effects on basal and hypoglycemic patterns of GPbb and GPmm expression were observed at specific VMN levels. LZ correspondingly down- or up-regulated baseline pyruvate recycling pathway marker protein expression in males (GLT) and females (ME-1), and altered hypoglycemic regulation of these proteins. Results document neuroestradiol regulation of VMN metabolic transmitter signaling of hypoglycemic energy deficiency in each sex. Sex-dimorphic GP variant protein expression and reactivity to aromatase may correlate with differential glycogen mobilization during hypoglycemia. Neuroestradiol may also exert sex-contingent control of glucogenic amino acid energy yield via action on distinctive enzyme targets in the male versus female.

Results provide novel proof that, in each sex, aromatase enzyme activity enhances basal aromatase protein expression and is required for hypoglycemic regulation of this protein profile in distinct rostro-caudal segments of the VMN. Evidence here for aromatase protein down- or up-regulation in the rostral versus middle/caudal male rat VMN infers that neuroestradiol signaling during IIH may be correspondingly diminished or augmented in rostral against other levels of this nucleus. Thus, hypoglycemia plausibly amplifies neuroestradiol transmitter volume in common as well as dissimilar regions of the VMN in the two sexes, as female rats exhibit, unlike males, amplified aromatase protein in the rostral and middle VMN. High levels of VMN aromatase expression support the likelihood that observable drug effects on VMN protein endpoints reflect in part direct action on local enzyme activity. However, since current studies involved Lz delivery by intra-LV infusion, the possibility that demonstrable effects of Lz treatment on, including aromatase protein content, may reflect in part drug suppression of enzyme activity both within and external to the VMN, including upstream neural loci that regulate VMN aromatase expression through afferent innervation cannot be discounted. Present work did not evaluate Lz treatment effects on segment-specific VMN tissue aromatase activity and estradiol content as quantitative methods of requisite sensitivity for measurement of these parameters in small-volume tissue samples collected from region-based VMN microdissection are not currently available. Outcomes identify a critical need to characterize the molecular mechanisms that achieve segment-specific effects of hypoglycemia on VMN aromatase protein profiles in each sex remain unclear. As gonadal steroids control brain aromatase gene expression and enyzme activity over the life span [27, 28, 34], it is possible that ERs may mediate, in part, distinctive aromatase responses to this metabolic stress in neuroanatomically-defined nerve cell populations.

Current research uniquely implicates neuroestradiol signaling in IIH patterns of VMN gluco-stimulatory NO and gluco-inhibitory GAD neurotransmission. Results support involvement, in each sex, of neuroestradiol-dependent and -independent mechanisms, discernible in specific rostro-caudal VMN segments, in hypoglycemic augmentation of VMN nNOS profiles. Lz was likewise observed to forestall hypoglycemic diminution of VMN GAD protein in some (male) or all (female) segments of the VMN. Additional effort will be needed to determine if hypoglycemia-associated adjustments in neuroestradiol production actively elicit changes in these transmitter marker protein profiles, or alternatively, if this local hormone stimulus is a passive requirement, irrespective of signal volume, for NO and GABA transmitter responses to hypoglycemia. The former scenario is supported by observations that, in each sex, regional patterns of hypoglycemic up-regulation of aromatase protein overlap, with a single exception, with aromatase activity-dependent nNOS augmentation and GAD suppression. Current data advance the novel premise that neuroestradiol may act as a critical acute transmitter targeting VMN glucoregulatory neuron substrates. The current consensus is that brain aromatase is expressed primarily in neurons [2, 13]. It would be informative to learn if this enzyme is present in VMN nitrergic and/or GABAergic nerve cells, and to understand if and how hypoglycemic regulation of this enzyme protein profile may affect intracellular estradiol content and receptor signaling in each cell type.

Current data indicate that forebrain aromatase activity up- or down-regulates baseline VMN GS protein expression in a rostro-caudal pattern that is unique to each sex. Here, IIH was observed to selectively suppress GS protein in dissimilar segments of the male (middle) versus female (caudal) VMN, an action that presumably curbs glycogen amassment during hypoglycemia in a sex-dimorphic manner. Outcomes further infer that aromatase activity is required only in the latter sex for this inhibitory GS response. Neuroestradiol signaling is also a putative positive stimulus for GPmm protein expression in distinctive regions of the male (rostral segment) versus female (rostral and middle segments) VMN. These results infer that aromatase signaling may facilitate NE stimulation of glycogen mobilization in those distinctive locations under euglycemic conditions. Data here document unique effects of hypoglycemia on GPmm profiles in each sex, as this GP variant was down-regulated in rostral and caudal levels of the male VMN, but instead declined in each analyzed segment of the female VMN. Hypoglycemic suppression of this NE-responsive GP variant likely reduces stimulation of glycogen breakdown by that neurotransmitter under glucoprivation versus glucostasis. In male rats, hypoglycemic suppression of GPmm likely involves neurosteroid-dependent (rostral VMN) and -independent (caudal VMN) mechanisms; similarly, GPmm diminution in the female rat VMN was both Lz-reversible (rostral and caudal VMN) and -refractory (middle VMN). At the same time, aromatase activity had a comparably minimal effect on baseline VMN GPbb expression in each sex. Moreover, GPbb profiles were affected differently by IIH compared to GPmm. For example, male rats showed elevated GPbb protein in all three VMN segments, whereas females had augmented GPbb expression in the rostral and caudal VMN yet decreased GPbb levels in the middle VMN. Hypoglycemic up-regulation of this energy-sensitive GP variant is postulated to expedite glycogen mobilization during glucose deficiency. Data here suggest that glycogen disassembly may be restricted to discrete levels of the female VMN under such conditions. Importantly, current outcomes implicate neuroestradiol signaling in hypoglycemic patterns of GPbb expression at all levels of the VMN in each sex.

Reports on differential cultured astrocyte GPmm and GPbb protein reactivity to distinctive physiological stimuli, i.e. NE versus AMP, support a role for these variants in stimulus-specific regulation of glycogen metabolism [24]. Proof here for dissimilar forebrain aromatase regulation of VMN GPmm versus GPbb protein in each sex infers that neuroestradiol shapes adjustments in glycogen metabolism during energy homeostasis versus imbalance. Outcomes here affirm new studies that document divergent adjustments in VMN GPmm (down-regulated) versus GPbb (up-regulated) profiles in response to hindbrain lactoprivation owing to pharmacological inhibition of local monocarboxylate transporter function [7]. NE links hindbrain dorsal vagal complex metabolic-sensory A2 noradrenergic neurons with the VMN and other hypothalamic gluco-regulatory loci. A2 neurons are a plausible site of detection of energetic sequelae of hypoglycemia as deficits in the oxidizable glycolytic endproduct l-lactate activate the ultra-sensitive energy sensor adenosine 5′-monophosphate-activated protein kinase (AMPK) in these cells during hypoglycemia, alongside augmentation of hypothalamic norepinephrine levels [30]. Hindbrain lactoprivic regulation of VMN GP variant protein expression involves NE. Additional research is warranted to examine whether hindbrain lactoprivic transmitter signals, namely NE, control VMN aromatase protein expression and corresponding neuroestradiol yield during hypoglycemia. There is also need to elucidate the mechanisms that impose rare-instance bi-directional neuroestradiol regulatory effects on VMN protein endpoints during eu- versus hypoglycemia, including the possibility that opposing actions may mediated by adjustments in steroid signal volume.

A notable outcome of current research is the discovery of sex-dimorphic baseline VMN GS and GP variant protein expression in the rat. Results indicate that basal VMN GS content varied significantly between sexes, with males exhibiting relatively higher (rostral and middle VMN) or lower (caudal) expression according to region. Interestingly, male rats showed higher baseline GPmm profiles in the rostral and caudal VMN, but greater middle VMN GPbb content compared to females. These data infer that amassed VMN glycogen in males may be more sensitive to NE- or glucoprivic-mediated glycogenolysis versus the female, depending upon VMN segment.

Data here document minimal neuroestradiol involvement in baseline VMN GLT protein expression in each sex, but reveal a stimulatory (rostral VMN) or inhibitory (middle and caudal VMN) influence of this stimulus on basal ME-1 profiles in female rats. Thus, in the female, aromatase activity may enhance or tamper utilization of glucogenic amino acids for energy production according to discrete VMN segment during energy homeostasis. VMN GLT profiles were increased by IIH in a region-specific manner unique to each sex; amplification of this pyruvate recycling pathway marker protein involved aromatase activity in male, but not female rats. It remains to be determined, for that sex, if differential neuroestradiol regulation of GLT during eu- versus hypoglycemia involves amplified steroid signaling and/or altered receptivity to that cue. Hypoglycemia augmented ME-1 protein expression, by neuroestradiol-independent mechanisms, in rostral and middle levels of the male VMN; in females, this protein was refractory to IIH. Taken together, these results infer that metabolism of non-glucose energy substrates may be restricted to distinct segments of the male VMN during hypoglycemia, and that sex-contingent VMN ME-1 reactivity to this metabolic stress may result in differential activity of the pyruvate recycling pathway in those regions.

## Conclusion

Results, summarized in Table 4, infer that neuroestradiol is required in each sex for optimal VMN metabolic transmitter signaling of hypoglycemic energy deficiency. Sex differences in VMN GP variant protein levels and sensitivity to aromatase may correlated with sex-dimorphic glycogen mobilization during this metabolic stress. Neuroestradiol may also exert sex-specific effects on glucogenic amino acid energy yield by actions on distinctive enzyme targets in each sex.

**Table 4:**
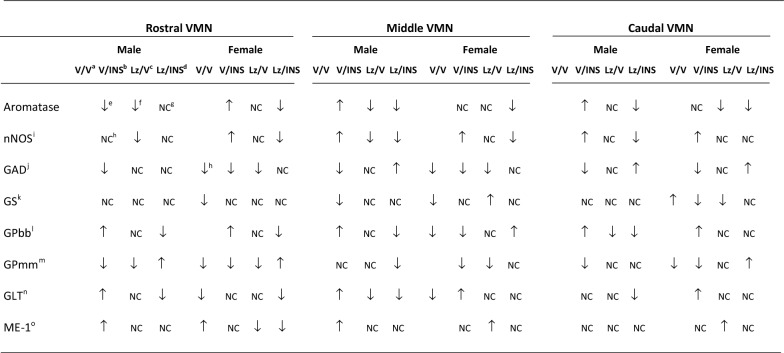
Summary of Rostro-Caudal patterns of aromatase control of ventromedial hypothalamic nucleus (VMN) metabolic transmitter, glycogen metabolic, and pyruvate recycling pathway enzyme protein expression in eu- and hypoglycemic male versus female rats

^a^ Intracerebroventricular (*icv*) vehicle (V) infusion days 5–11; subcutaneous (*sc*) vehicle (V) injection day 11

^b^ *icv* V infusion days 5–11; *sc* 10.0 U neutral protamine Hagedorn insulin (INS)/kg bw injection day 11

^c^ *icv* letrozole (1.67 μg/μL dosage, 0.5 μL/h infusion rate) infusion days 5–11; sc V injection day 11

^d^ *icv* letrozole infusion days 5–11; *sc* INS injection day 11

^e^ V/INS vs V/V

^f^ Lz/V vs V/V

^g^ Lz/INS vs Lz/V

^h^ No change

^i^ Neuronal nitric oxide synthase

^j^ Glutamate decarboxylase 65/67

^k^ Glycogen synthase

^l^ Glycogen phosphorylase-brain type

^m^ Glycogen phosphorylase-muscle type

^n^ Glutaminase

^o^ Malic enzyme-1

## Supplementary information

**Additional file 1.** Panels 1–8 depict full uncropped Western blots that correspond to cropped images from those blots that are presented in Figs. 1, 2, 3, 4, 5, 6, 7 and 8, respectively.

## Data Availability

A statement is included in the manuscript reads as follows: ‘All data generated during and/or analyzed during the current study are available from the corresponding author on reasonable request.

## Abbreviations

GABA: γ-Aminobutyric acid
GAD: Glutamate decarboxylase_65/67_
GLT: Glutaminase
INS: Insulin
IIH: Insulin-induced hypoglycemia
LZ: Letrozole
ME-1: NADP-dependent malic enzyme-1
nNOS: Neuronal nitric oxide synthase
OVX: Ovariectomy
VMN: Ventromedial hypothalamic nucleus
