# Supplementary figures and images for: Sex-dimorphic neuroestradiol regulation of ventromedial hypothalamic nucleus glucoregulatory transmitter and glycogen metabolism enzyme protein expression in the rat
Source: BMC Neurosci. 2020 Nov 25;21:51. doi: 10.1186/s12868-020-00598-w (PMC7687823; doi:10.1186/s12868-020-00598-w)

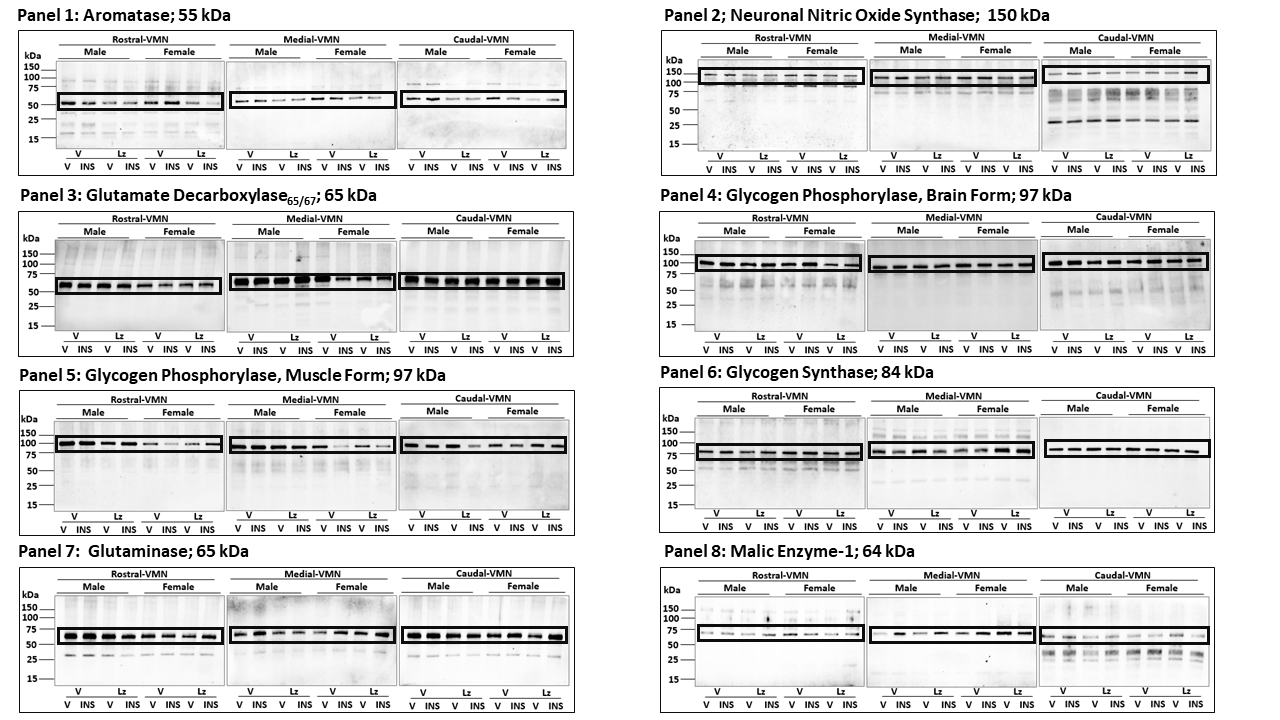

Supplement: Supplementary file 1 — Additional file 1. Panels 1–8 depict full uncropped Western blots that correspond to cropped images from those blots that are presented in Figs. 1, 2, 3, 4, 5, 6, 7 and 8, respectively. [file 12868_2020_598_MOESM1_ESM.tif]
